# Supplementary material for: SILAC-Based Characterization of Plasma-Derived Extracellular Vesicles in Patients Undergoing Partial Hepatectomy
Source: Int J Mol Sci. 2024 Oct 4;25(19):10685. doi: 10.3390/ijms251910685 (PMC11476990; doi:10.3390/ijms251910685)
Supplement: Supplementary file 1 [file ijms-25-10685-s001.zip › Supl Table 1.pdf]

SUPPLEMENTAL MATERIAL

SILAC-based characterization of plasma-derived extracellular vesicles in patients undergoing partial hepatectomy

Ulrike Resch[1,2]*, Hubert Hackl[3], David Pereyra[4], Jonas Santol[4], Laura Brunnthaler[1], Joel Probst[4], Anna Sofie Jankoschek[4], Monika Aiad[4], Hendrik Nolte[2], Marcus Krueger[2], Patrick Starlinger[34,5], Alice Assinger[1]*

1) Supplemental Table

1) Supplementary Table

Parameter		Cohort (n=9)	PHLF Cohort (n=3)	no PHLF Cohort (n=3)	ALPPS Cohort (n=3)	Missing values
Sex	Male	7 (77.8%)	3 (100%)	2 (66.6%)	2 (66.6%)	
	Female	2 (22.2%)	0 (0.0%)	1 (33.3%)	1 (33.3%)	
Age (y, range)		64.9 (50.0-78.6)	65.3 (56.5-76.0)	58.6 (50.0-69.6)	70.9 (56.5-78.6)	
Hepatic resection	Minor (< 3 segments)	2 (22.2%)	0 (0.0%)	0 (0.0%)	2 (66.6%)	
	Major (≥ 3 segments)	7 (77.8%)	3 (100.0%)	3 (100.0%)	1 (33.3%)	
**Hepatic comorbidities***						
	Steatosis (%)	14.0 (0.0-60.0%)	33.0 (0.0-60.0%)	8 (0.0-20.0%)	0 (0.0-0.0%)	
	Steatohepatitis	3 (33.3%)	2 (66.6%)	1 (33.3%)	0 (0.0)	
	Fibrosis grade (mean)	1	1	1	1	2 (22.2%)
	CASH	3 (33.3%)	2 (66.6%)	1 (33.3%)	0 (0.0)	1 (2.1%)
Etiology of underlying liver disease						
	No underlying liver disease	6	1	2	3	
	MetALD	3	2	1	0	
**Preoperative parameters**						
	PDR (%)	20.5 (15.0-30.0)	18.6 (17.7-19.4)	23.7 (18.0-30.0)	15.0 (15.0-15.0)	3 (33.3%)
	Platelet counts (×10³/µL)	221 (178-267)	199 (178-206)	237 (234-239)	233 (201-267)	1 (11.1%)
	SB (mg/dL) mean (range)	0.8 (0.3 – 2.9)	1.5 (0.4 – 2.9)	0.5 (0.3-0.6)	0.4 (0.3-0.6)	1 (11.1%)
	PT (%) mean (range)	98 (45 – 120)	76 (45 – 98)	108 (106-110)	114 (109-120)	1 (11.1%)
	AP (U/L) mean (range)	96 (64 – 128)	67 (64 – 68)	108 (90 – 126)	117 (98-128)	1 (11.1%)
	GGT (U/L) mean (range)	68 (33– 112)	51 (33-74)	74 (36 – 112)	81 (66-88)	1 (11.1%)
	AST (U/L) mean (range)	42 (25 – 71)	57 (43 – 71)	37 (27 – 46)	25 (25-25)	4 (44.4%)
	ALT (U/L) mean (range)	38 (20 – 81)	54 (30 – 81)	39 (37 – 41)	22 (20-25)	1 (11.1%)
	Albumin (g/L) mean (range)	41.8 (37.3 – 47.2)	42.3 (37.3 – 47.2)	40.8 (40.8-40.8)	n.a.	6 (66.6%)
**Morbidity**						
	No morbidity	5 (55.5%)	3 (100.0%)	2 (66.6%)	0 (0.0%)	

	Grade I	1 (11.1%)	0 (0.0%)	1 (33.3%)	0 (0.0%)	
	Grade II	2 (22.2%)	0 (0.0%)	0 (0.0%)	2 (66.6%)	
	Grade III	1 (11.1%)	0 (0.0%)	0 (0.0%)	1 (33.3%)	
	Grade IV	0 (0.0%)	0 (0.0%)	0 (0.0%)	0 (0.0%)	
	Grade V	0 (0.0%)	0 (0.0%)	0 (0.0%)	0 (0.0%)	
**Postoperative stay**						
	ICU (days)	1.0 (0.0-2.0)	1.0 (1.0-2.0)	1.0 (0.0-1.0)	1 (0.0-2.0)	
	Total hospitalization (d)	11 (7-22)	8 (6-10)	9 (7-14)	22 (7-22)	
**PHLF ISGLS**						
	no PHLF	6 (66.6%)	0 (0.0%)	3 (100.0%)	3 (100.0%)	
	Grade A	1 (11.1%)	1 (33.3%)	0 (0.0%)	0 (0.0%)	
	Grade B	2 (22.2%)	2 (66.6%)	0 (0.0%)	0 (0.0%)	
	Grade C	0 (0.0%)	0 (0.0%)	0 (0.0%)	0 (0.0%)	

Table 1: Plasma cohort patient characteristics: Hepatic comorbities*, all patients received Neoadjuvant chemotherapy; y, years; d, days; ALT, alanine aminotransferase; AP, alkaline phosphatase; AST, aspartate aminotransferase; CASH, chemotherapy-induced acute steatohepatitis; CCC, cholangiocellular carcinoma; GGT, gamma-glutamyl transpeptidase; HCC, hepatocellular carcinoma; ICU, intensive care unit; ISGLS, International Study Group of Liver Surgery; mCRC, metastatic colorectal cancer; PHLF, post-hepatectomy liver failure; PDR, plasma disappearance rate; PT, prothrombin time; SB, serum bilirubin.
