# Supplementary material for: SILAC-Based Characterization of Plasma-Derived Extracellular Vesicles in Patients Undergoing Partial Hepatectomy
Source: Int J Mol Sci. 2024 Oct 4;25(19):10685. doi: 10.3390/ijms251910685 (PMC11476990; doi:10.3390/ijms251910685)
Supplement: Supplementary file 1 [file ijms-25-10685-s001.zip › SUPPLEMENTAL MATERIAL IJMS_SI_Characterization of Extracellular Vesicles in Disease_Resch.pdf]

SUPPLEMENTAL MATERIAL

SILAC-based characterization of plasma-derived extracellular vesicles in patients undergoing partial hepatectomy

Ulrike Resch[1,2]*, Hubert Hackl[3], David Pereyra[4], Jonas Santol[4], Laura Brunnthaler[1], Joel Probst[4], Anna Sofie Jankoschek[4], Monika Aiad[4], Hendrik Nolte[2], Marcus Krueger[2], Patrick Starlinger[34,5], Alice Assinger[1]*

1) Supplemental Table

2) Supplementary Figures

3) Description to supplemental data

1) Supplementary Table S1

Parameter		Cohort (n=9)	PHLF Cohort (n=3)	no PHLF Cohort (n=3)	ALPPS Cohort (n=3)	Missing values
Sex	Male	7 (77.8%)	3 (100%)	2 (66.6%)	2 (66.6%)	
	Female	2 (22.2%)	0 (0.0%)	1 (33.3%)	1 (33.3%)	
Age (y, range)		64.9 (50.0-78.6)	65.3 (56.5-76.0)	58.6 (50.0-69.6)	70.9 (56.5-78.6)	
Hepatic resection	Minor (< 3 segments)	2 (22.2%)	0 (0.0%)	0 (0.0%)	2 (66.6%)	
	Major (≥ 3 segments)	7 (77.8%)	3 (100.0%)	3 (100.0%)	1 (33.3%)	
**Hepatic comorbidities***						
	Steatosis (%)	14.0 (0.0-60.0%)	33.0 (0.0-60.0%)	8 (0.0-20.0%)	0 (0.0-0.0%)	
	Steatohepatitis	3 (33.3%)	2 (66.6%)	1 (33.3%)	0 (0.0)	
	Fibrosis grade (mean)	1	1	1	1	2 (22.2%)
	CASH	3 (33.3%)	2 (66.6%)	1 (33.3%)	0 (0.0)	1 (2.1%)
Etiology of underlying liver disease						
	No underlying liver disease	6	1	2	3	
	MetALD	3	2	1	0	
**Preoperative parameters**						
	PDR (%)	20.5 (15.0-30.0)	18.6 (17.7-19.4)	23.7 (18.0-30.0)	15.0 (15.0-15.0)	3 (33.3%)
	Platelet counts (×10³/μL)	221 (178-267)	199 (178-206)	237 (234-239)	233 (201-267)	1 (11.1%)
	SB (mg/dL) mean (range)	0.8 (0.3 – 2.9)	1.5 (0.4 – 2.9)	0.5 (0.3-0.6)	0.4 (0.3-0.6)	1 (11.1%)
	PT (%) mean (range)	98 (45 – 120)	76 (45 – 98)	108 (106-110)	114 (109-120)	1 (11.1%)
	AP (U/L) mean (range)	96 (64 – 128)	67 (64 – 68)	108 (90 – 126)	117 (98-128)	1 (11.1%)
	GGT (U/L) mean (range)	68 (33– 112)	51 (33-74)	74 (36 – 112)	81 (66-88)	1 (11.1%)
	AST (U/L) mean (range)	42 (25 – 71)	57 (43 – 71)	37 (27 – 46)	25 (25-25)	4 (44.4%)
	ALT (U/L) mean (range)	38 (20 – 81)	54 (30 – 81)	39 (37 – 41)	22 (20-25)	1 (11.1%)
	Albumin (g/L) mean (range)	41.8 (37.3 – 47.2)	42.3 (37.3 – 47.2)	40.8 (40.8-40.8)	n.a.	6 (66.6%)
**Morbidity**						
	No morbidity	5 (55.5%)	3 (100.0%)	2 (66.6%)	0 (0.0%)	

	Grade I	1 (11.1%)	0 (0.0%)	1 (33.3%)	0 (0.0%)	
	Grade II	2 (22.2%)	0 (0.0%)	0 (0.0%)	2 (66.6%)	
	Grade III	1 (11.1%)	0 (0.0%)	0 (0.0%)	1 (33.3%)	
	Grade IV	0 (0.0%)	0 (0.0%)	0 (0.0%)	0 (0.0%)	
	Grade V	0 (0.0%)	0 (0.0%)	0 (0.0%)	0 (0.0%)	
**Postoperative stay**						
	ICU (days)	1.0 (0.0-2.0)	1.0 (1.0-2.0)	1.0 (0.0-1.0)	1 (0.0-2.0)	
	Total hospitalization (d)	11 (7-22)	8 (6-10)	9 (7-14)	22 (7-22)	
**PHLF ISGLS**						
	no PHLF	6 (66.6%)	0 (0.0%)	3 (100.0%)	3 (100.0%)	
	Grade A	1 (11.1%)	1 (33.3%)	0 (0.0%)	0 (0.0%)	
	Grade B	2 (22.2%)	2 (66.6%)	0 (0.0%)	0 (0.0%)	
	Grade C	0 (0.0%)	0 (0.0%)	0 (0.0%)	0 (0.0%)	

2) Supplementary Figures

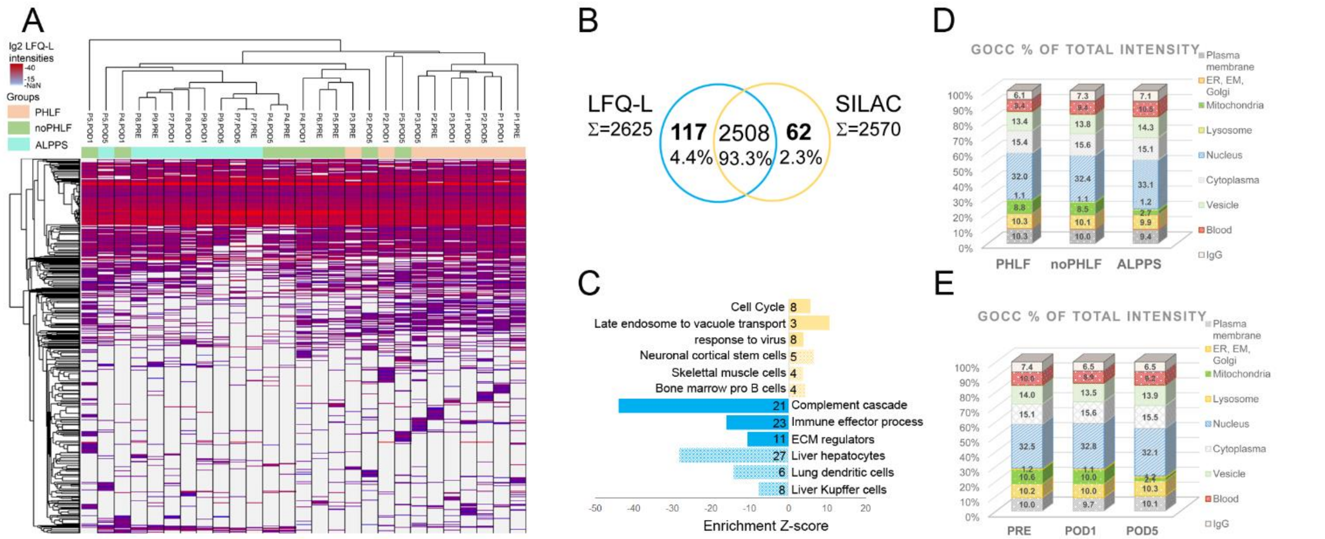

Supplementary Figure 1: Qualitative comparison of LFQ-L and SILAC identifications to capture plasma specific proteins: **A)** Heatmap of hierarchical clustered samples (average linked Euclidian distance on log2 transformed LFQ-L intensities, missing values in light grey) to visualize data completeness across sample groups PHLF, noPHLF and ALPPS as indicated. **B)** Venn diagram of common and unique proteins identified on the basis of LFQ-L and SILAC-ratios, **C)** Enriched Gene Ontology and cell type signatures of 117 LFQ-L (blue) or 62 SILAC-only proteins (yellow) with respective protein numbers denoted. **D)** Overall GOCC categorization of all quantified proteins and their relative contribution to the sum intensity (LFQ-L) in sample groups “outcome” (PHLF, noPHLF

and ALPPS) or “time” (before surgery, 1 and 5 days after PHx). Corresponding source data can be found in Suppl. Data 1.

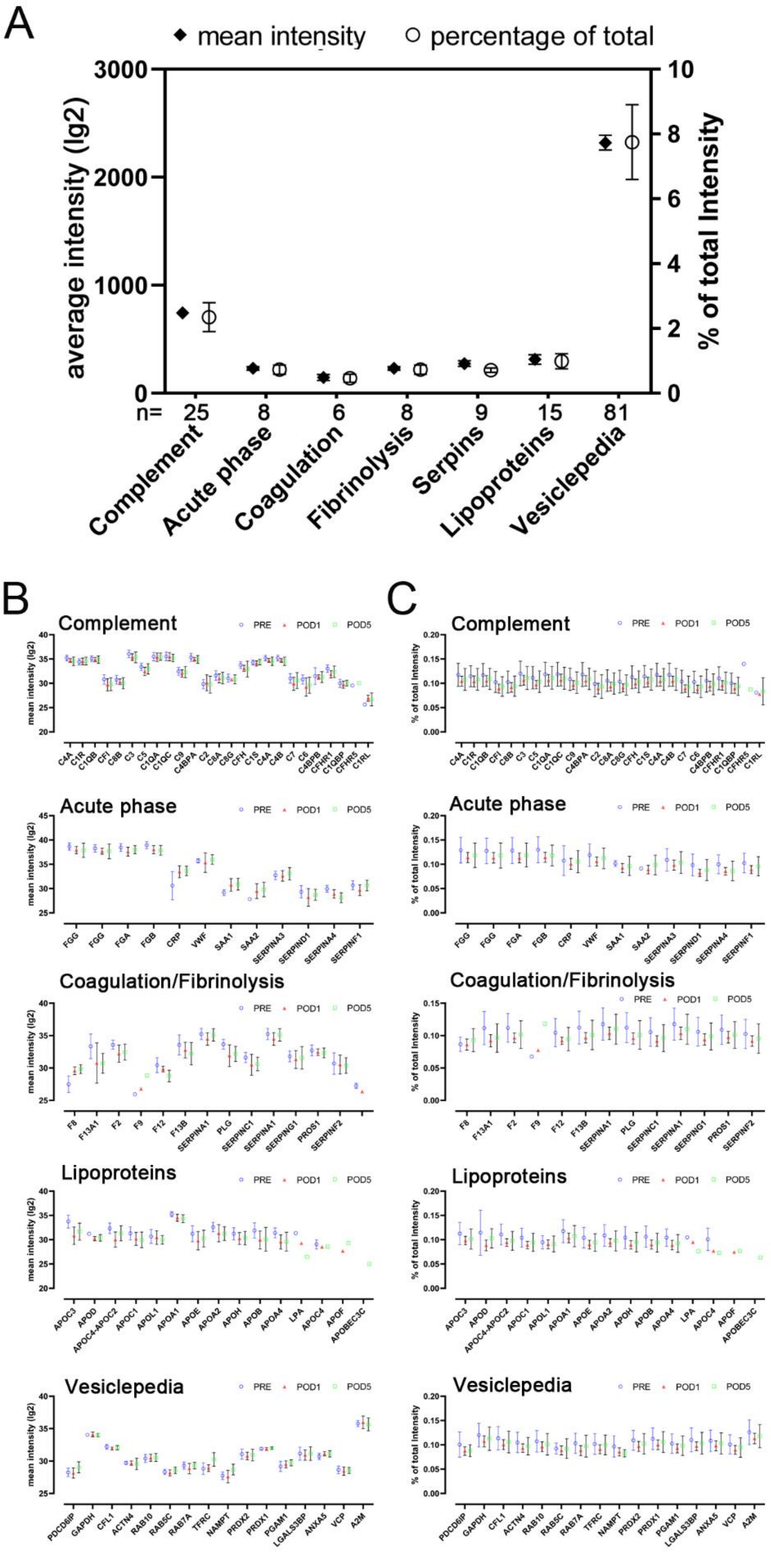
A
mean intensity
percentage of total
average intensity (lg2)
% of total Intensity
n=
25
8
6
8
9
15
81
Complement
Acute phase
Coagulation
Fibrinolysis
Serpins
Lipoproteins
Vesiclepedia
B
C
Complement
Acute phase
Coagulation/Fibrinolysis
Lipoproteins
Vesiclepedia
PRE
POD1
POD5
mean intensity (lg2)
% of total Intensity
C4A
C1R
C1QB
CFI
C8B
C3
C5
C1QA
C1QC
C9
C4BPA
C2
C8A
C8G
CFH
C1S
C4A
C4B
C7
C6
C4BPB
CFHR1
C1QBP
CFHR5
C1RL
FGG
FGG
FGA
FGB
CRP
VWF
SAA1
SAA2
SERPINA3
SERPIND1
SERPINA4
SERPINF1
F8
F13A1
F2
F9
F12
F13B
SERPINA1
PLG
SERPINC1
SERPINA1
SERPING1
PROS1
SERPINF2
APOC3
APOD
APOC4-APOC2
APOC1
APOL1
APOA1
APOE
APOA2
APOH
APOB
APOA4
LPA
APOC4
APOF
APOBEC3C
PDCD6IP
GAPDH
CFL1
ACTN4
RAB10
RAB5C
RAB7A
TFRC
NAMPT
PRDX2
PRDX1
PGAM1
LGALS3BP
ANXA5
VCP
A2M

Supplementary Figure 2: Blood- and Vesiclepedia proteins identified and quantified in plasma EVs: Cumulative **(A)** and individual **(B, C)** abundance of blood (complement, acute phase, coagulation fibrinolysis, serpins (serine-protease inhibitor family members), lipoproteins and selected EV-proteins (Vesiclepedia) quantified in EV-samples at three different time points (before surgery, on postoperative day 1 and 5) with respective protein counts in each category denoted on the x-axis. Data are expressed as mean±sd log2-transformed LFQ-L intensity values **(B)** or percentage of total intensities **(C)** in merged outcome groups. Corresponding source data can be found in Suppl. Data 1.

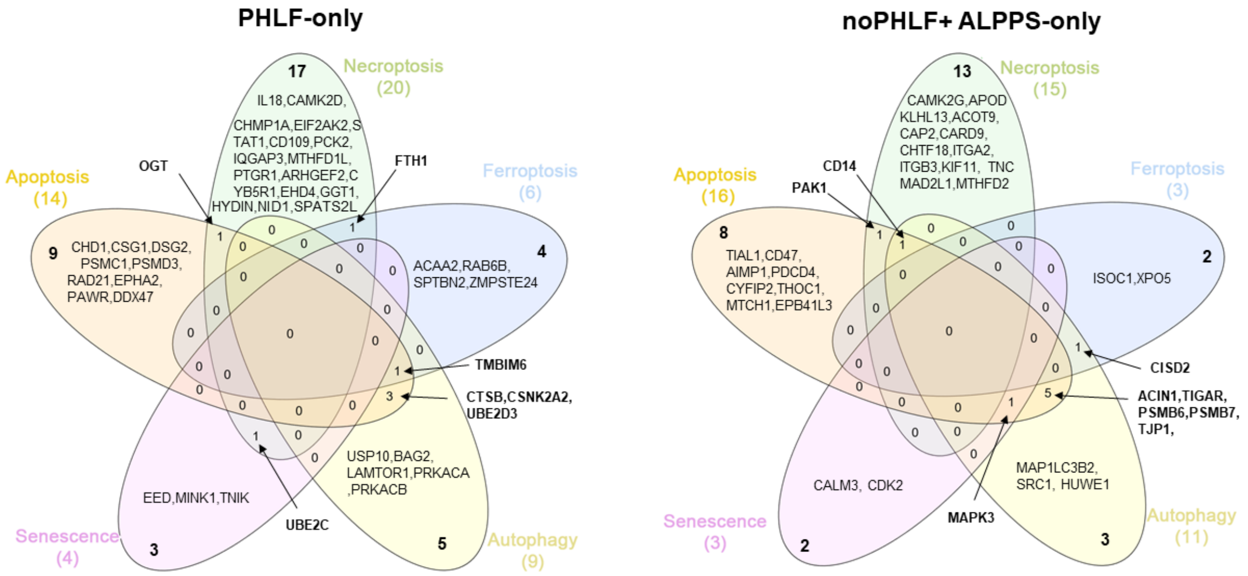

Supplementary Figure 3: EV-proteins with functions in stress response and homeostasis. Venn diagrams of proteins with functions in cell death (apoptosis, necroptosis and ferroptosis), auto(mito)phagy and senescence identified in 255 PHLF or 198 noPHLF + ALPPS only identified at least 3 times in outcome groups across all three time points (before surgery, on postoperative day 1 and 5).

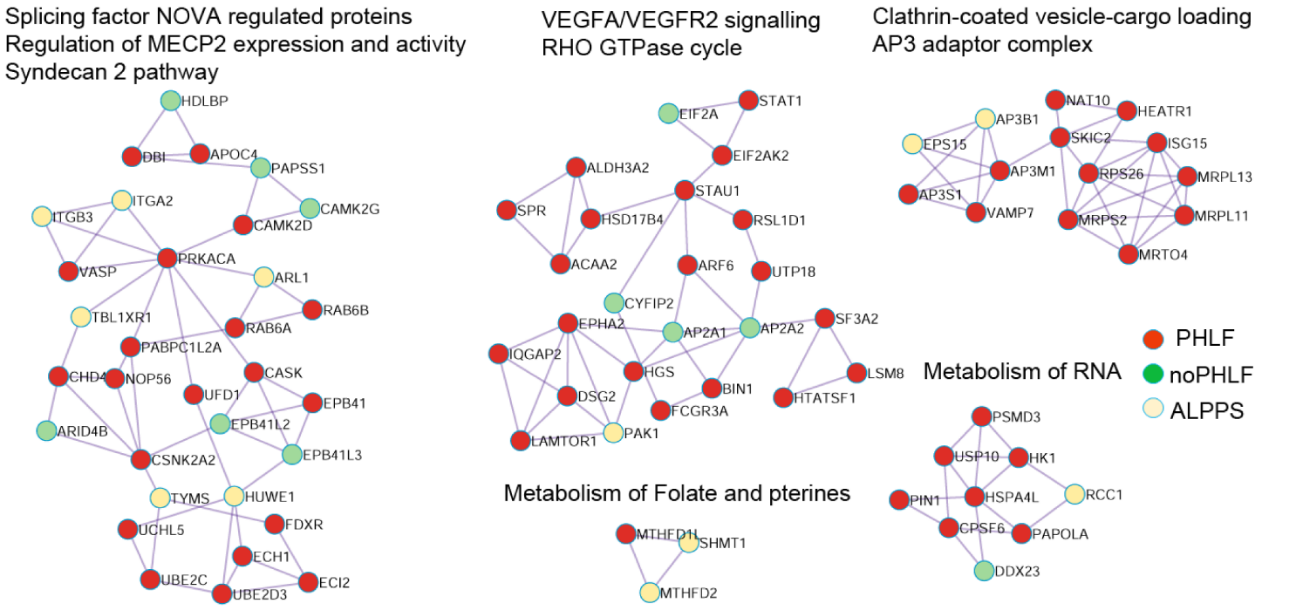

Network	Annotation
_FINAL	R-HSA-8953854|Metabolism of RNA|-15.3;R-HSA-6798695|Neutrophil degranulation|-12.2;GO:0055086|nucleobase-containing small molecule metabolic process|-11.1
FINAL MCODE_ALL	WP3888|VEGFA VEGFR2 signaling|-8.6;GO:0035999|tetrahydrofolate interconversion|-8.2;GO:0022613|ribonucleoprotein complex biogenesis|-8.0
_FINAL_SUB1 _MCODE_1	WP4148|Splicing factor NOVA regulated synaptic proteins|-9.1;R-HSA-9022692|Regulation of MECP2 expression and activity|-7.4;M240|PID SYNDECAN 2 PATHWAY|-7.4
_FINAL_SUB1 _MCODE_2	WP3888|VEGFA VEGFR2 signaling|-8.9;R-HSA-9012999|RHO GTPase cycle|-8.7;R-HSA-194315|Signaling by Rho GTPases|-7.2
_FINAL_SUB1 _MCODE_3	GO:0035654|clathrin-coated vesicle cargo loading, AP-3-mediated|-8.6;GO:0035652|clathrin-coated vesicle cargo loading|-8.6;CORUM:59|AP3 adaptor complex|-8.6
_FINAL_SUB1 _MCODE_4	R-HSA-8953854|Metabolism of RNA|-4.4;R-HSA-72203|Processing of Capped Intron-Containing Pre-mRNA|-4.2;GO:0006397|mRNA processing|-3.5
_FINAL_SUB1 _MCODE_5	GO:0035999|tetrahydrofolate interconversion|-10.7;R-HSA-196757|Metabolism of folate and pterines|-9.8;GO:0046653|tetrahydrofolate metabolic process|-9.7

created by http://metascape.org

Supplementary Figure 4: Enriched protein-protein-interaction (PPI) network modules in EVs: **A)** Combined PPI network module enrichment analysis of proteins identified only in PHLF (225), noPHLF (88) or ALPPS (70) independent of sampling time after filtering LFQ-L identifications for at least 3 valid values as depicted in Venn diagram Fig. 2C. **B)** Enrichment-term-table with corresponding p-values (log10) on overall (_FINAL_MCODE_ALL) and sub-network modules (_FINAL_SUB1_MCODE 1-5). Analysis was performed in Metascore using the "multiple gene list"-option (Suppl. Data 2) with default settings and networks were color coded in Cytoscape.

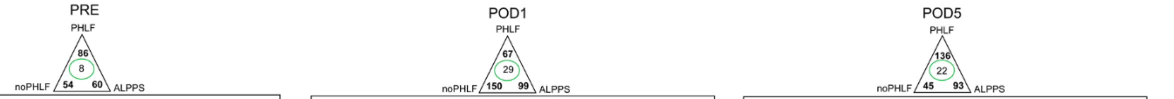

Top 5 "only" proteins in PHLF

	Gene Symbol	Protein name	Protein ID
PRE	GIGYF2	PERQ amino acid-rich with GYF domain-containing protein 2	Q6Y7W6
	DNAJB13	DnaJ homolog subfamily B member 13	P59910
	PPP1R7	Protein phosphatase 1 regulatory subunit 7	Q15435
	CLIP1	CAP-Gly domain-containing linker protein 1	P30622
	F12	Coagulation factor XII	P00748
POD1	RNF40	E3 ubiquitin-protein ligase BRE1B	O75150
	L1TD1	LINE-1 type transposase domain-containing protein 1	Q5T7N2
	FAM50A	Protein FAM50A	Q14320
	MARS	Methionine--tRNA ligase, cytoplasmic	P56192
	HIST2H2AB	Histone H2A type 2-B	Q8IUE6
POD5	STXBP5	Syntaxin-binding protein 5	Q5T5C0
	MED24	Mediator of RNA polymerase II transcription subunit 24	O75448
	CHD4	Chromodomain-helicase-DNA-binding protein 4	Q14839
	PIGR	Polymeric immunoglobulin receptor;Secretory component	P01833
	ZC3H15	Zinc finger CCCH domain-containing protein 15	Q8WU90

Top 5 "only" proteins in noPHLF

	Gene Symbol	Protein name	Protein ID
PRE	ATP11A	Probable phospholipid-transporting ATPase IH	P98196
	KEAP1	Kelch-like ECH-associated protein 1	Q14145
	MARS	Methionine--tRNA ligase, cytoplasmic	P56192
	KLKB1	Plasma kallikrein	P03952
	TTN	Titin	Q8WZ42
POD1	TRMT11	tRNA (guanine(10)-N2)-methyltransferase homolog	Q7Z4G4
	MED24	Mediator of RNA polymerase II transcription subunit 24	O75448
	PFDN1	Prefoldin subunit 1	O60925
	SSFA2	Sperm-specific antigen 2	P28290
	CUL2	Cullin-2	Q13617
POD5	ARID4B	AT-rich interactive domain-containing protein 4B	Q4LE39
	L1TD1	LINE-1 type transposase domain-containing protein 1	Q5T7N2
	MARK3	MAP/microtubule affinity-regulating kinase 3	P27448
	SUB1	Activated RNA polymerase II transcriptional coactivator p15	P53999
	LRPAP1	Alpha-2-macroglobulin receptor-associated protein	P30533

Top 5 "only" proteins in ALPPS

	Gene Symbol	Protein name	Protein ID
PRE	RPL24	60S ribosomal protein L24	P83731
	STXBP5	Syntaxin-binding protein 5	Q5T5C0
	MLLT4	Afadin	P55196
	AKR1B1	Aldose reductase	P15121
	MST1	Hepatocyte growth factor-like protein	P26927
POD1	FKBP5	Peptidyl-prolyl cis-trans isomerase FKBP5	Q13451
	RPLP2	60S acidic ribosomal protein P2	P05387
	DOCK7	Dedicator of cytokinesis protein 7	Q96N67
	HSD17B10	3-hydroxyacyl-CoA dehydrogenase type-2	Q99714
	FTH1	Ferritin heavy chain	P02794
POD5	DDOST	Dolichyl-diphosphooligosaccharide-protein glycosyltransferase	P39656
	GRAP	GRB2-related adapter protein	Q13588
	PFDN1	Prefoldin subunit 1	O60925
	RPLP1	60S acidic ribosomal protein P1	P05386
	STON2	Stonin-2	Q8WXE9

Supplementary Figure 5: Unique proteins in sampling time and outcome groups: Intersections of "only" proteins identified a least twice at each timepoint (prior to surgery, on postoperative day 1 and 5) in respective outcome groups (PHLF, noPHLF or ALPPS). Top 5 proteins (ranked on the basis of LFQ-L abundance) for each time point in respective outcome groups. Complete lists are provided in Suppl. Data 2.

A

Top 20 up and downregulated PHLF vs noPHLF

Gene Symbol	FC (lg2) UP PHLF	p-value	Gene Symbol	FC (lg2) UP noPHLF	p-value
DRAP1	1.86	0.004	UHRF1	-1.80	0.000
GGCT	1.78	0.009	CTSB	-1.75	0.006
NAA50	1.66	0.004	TIMM44	-1.74	0.003
SDHA	1.63	0.000	CSNK1A1	-1.72	0.046
AIMP1	1.62	0.035	VTN	-1.72	0.021
ATP1B3	1.61	0.005	AMBP	-1.59	0.007
NSUN2	1.60	0.021	PES1	-1.56	0.005
RAB13	1.55	0.002	RPL27A	-1.54	0.000
EPHX1	1.54	0.033	KATNAL2	-1.53	0.004
APOE	1.52	0.009	SMCHD1	-1.51	0.013
PPCS	1.50	0.001	CASK	-1.49	0.045
RAB3GAP2	1.47	0.042	RSL1D1	-1.49	0.030
BOLA2	1.43	0.038	NXN	-1.42	0.048
RAB8B	1.34	0.007	EEF1B2	-1.28	0.018
FAU	1.31	0.039	ATP2A2	-1.26	0.040
YWHAQ	1.30	0.002	LRRFIP2	-1.25	0.045
LONP1	1.30	0.048	FAM136A	-1.23	0.044
YWHAB	1.29	0.016	DSTN	-1.22	0.007
RNPEP	1.29	0.033	DNM1L	-1.20	0.012
SFN	1.29	0.003	NACA	-1.06	0.044

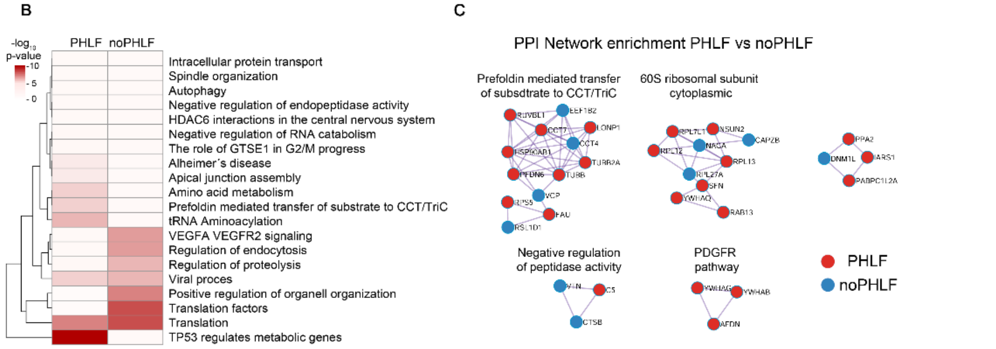

D

Top 20 up and downregulated PHLF vs ALPPS

Gene Symbol	FC (lg2) UP PHLF	p-value	Gene Symbol	FC (lg2) UP ALPPS	p-value
PSMB6	2.09	0.009	NUMA1	-2.24	0.003
SDHA	2.07	0.000	COPA	-2.03	0.000
APOE	2.05	0.004	CMBL	-1.99	0.000
SUMO1	1.95	0.002	GNAS	-1.94	0.019
IK	1.89	0.001	GMPS	-1.86	0.002
RPS15A	1.81	0.000	SF3B1	-1.85	0.000
SAFB2	1.80	0.011	SERPINB5	-1.83	0.011
HACD2	1.80	0.000	MGME1	-1.81	0.001
DBI	1.80	0.011	IGF2R	-1.70	0.026
LMNB2	1.78	0.041	NDUFS4	-1.70	0.001
CAPRIN1	1.67	0.001	VPS4A	-1.66	0.004
CPNE3	1.66	0.002	ASL	-1.66	0.042
IDH3A	1.64	0.000	EXOSC9	-1.64	0.000
TUBB3	1.61	0.000	TCEA1	-1.64	0.004
BCAS2	1.59	0.017	FKBP5	-1.63	0.036
HNRNPDL	1.58	0.000	DHRS4	-1.62	0.000
TUBB6	1.57	0.000	ASS1	-1.59	0.027
RAD21	1.56	0.046	SF1	-1.57	0.048
MDH1	1.56	0.002	CLTCL1	-1.54	0.027
LMAN2	1.55	0.017	PANK4	-1.53	0.005

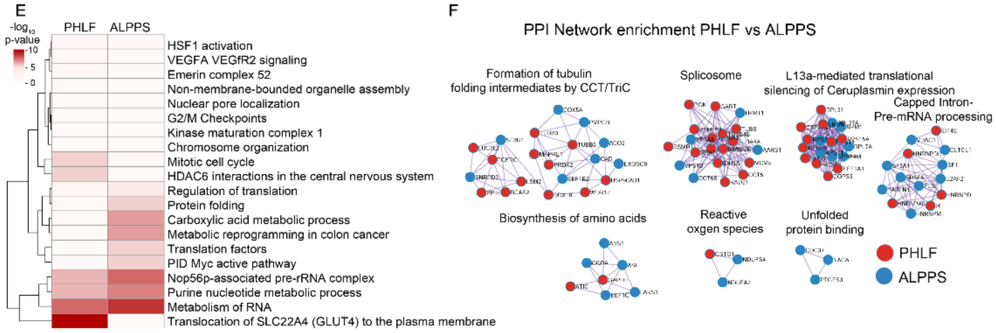

Supplementary Figure 6: EV-protein cargo signatures discriminating PHLF from noPHLF and ALPPS: **A-C**: Summary of pairwise comparison PHLF versus noPHLF-EVs. Top 20 up and downregulated EV-proteins (A), enriched GO-terms **(B)** and PPI Network modules **(C)** thereof. **D-F**: Summary of pairwise comparison PHLF versus ALPPS-EVs. Top 20 up and downregulated EV-proteins **(D)**, enriched GO-terms **(E)** and PPI Network modules **(F)**.

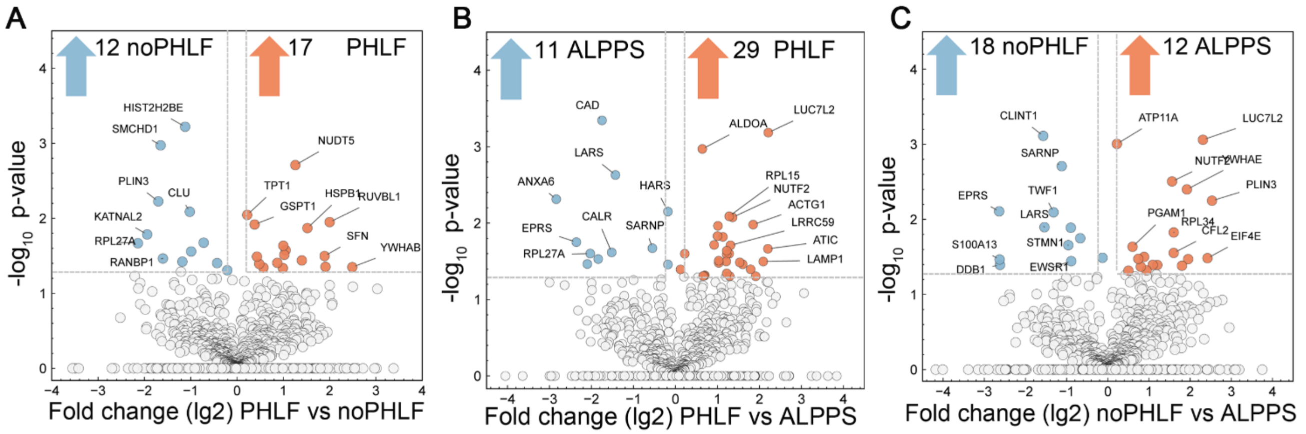

PHLF versus noPHLF

downregulated Gene Symbol	FC (lg2)	upregulated Gene Symbol	FC (lg2)
RPL27A	-2.14	YWHAB	2.49
KATNAL2	-1.94	RUVBL1	2.00
PLIN3	-1.70	IARS	1.91
SMCHD1	-1.65	SFN	1.89
RANBP1	-1.60	HSPB1	1.52
LDHA	-1.18	RPL13	1.40
HIST2H2BE	-1.12	NUDT5	1.26
CLU	-1.02	EWSR1	1.05
ETF1	-0.99	FBL	1.02
ACTN4	-0.72	YWHAQ	1.00

PHLF versus ALPPS

downregulated Gene Symbol	FC (lg2)	upregulated Gene Symbol	FC (lg2)
ANXA6	-2.84	LUC7L2	2.21
EPRS	-2.36	ATIC	2.20
SRSF5	-2.10	LAMP1	2.09
RPL27A	-2.03	CAPRIN1	1.91
TCEB1	-1.84	ACTG1	1.85
CAD	-1.75	HSPB1	1.79
CALR	-1.52	EEF1A1	1.60
LARS	-1.43	ESD	1.53
SARNP	-0.55	NUTF2	1.36
HARS	-0.18	LRRC59	1.31

noPHLF versus ALPPS

downregulated Gene Symbol	FC (lg2)	upregulated Gene Symbol	FC (lg2)
EPRS	-2.64	PLIN3	2.53
S100A13	-2.63	EIF4E	2.42
DDB1	-2.62	LUC7L2	2.31
CLINT1	-1.57	ATP5F1	1.95
LARS	-1.54	YWHAE	1.92
TWF1	-1.32	ILF3	1.80
SARNP	-1.12	RPL34	1.60
STMN1	-0.96	CFL2	1.60
HSPA1B	-0.90	NUTF2	1.56
EWSR1	-0.89	TUBB6	1.20

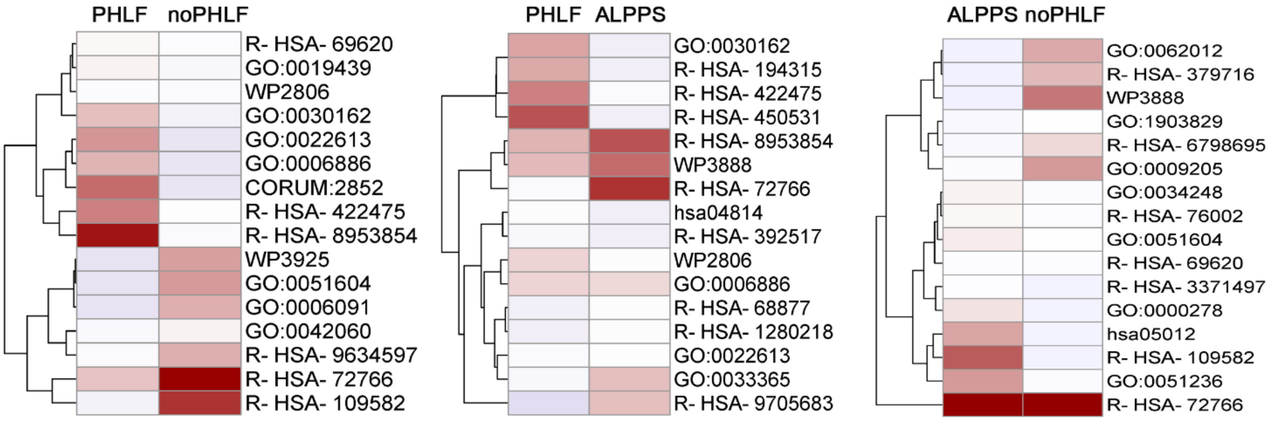

Supplementary Figure 7: PREdictive outcome signatures: prior to surgery: Comparative statistical analysis of EV proteins before PHx (PRE) in different outcome groups. Volcano plots, top 10 regulated proteins and GO-pathway enrichments analysis (including significantly changed and "only" proteins) in PHLF versus noPHLF **(A)** or PHLF versus ALPPS **(B)** or noPHLF versus ALPPS **(C)**. GO-term-names of A and B are presented in Fig.4 and complete source data can be found in Suppl. Data 4.

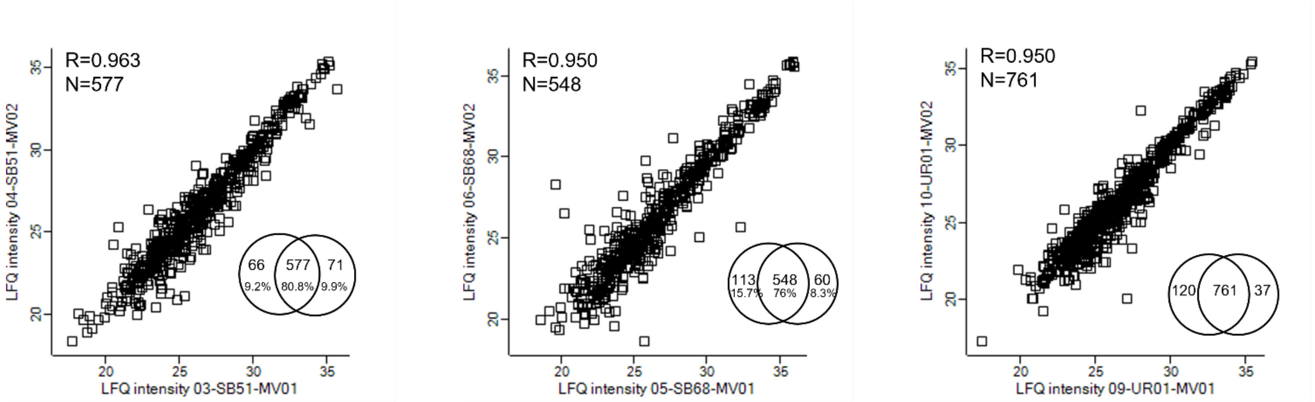

Supplementary Figure 8: Reproducibility of ultracentrifugation-based EV isolation: Plasma from 3 subjects (á 150µl in replica) were subjected to ultracentrifugation- based EV isolation and LC-MS/MS analysis as described in methods. Data were filtered for reverse, contaminants, LFQ intensity values lg2-transformed and replica plotted against each other. Venn diagram show identification overlaps and Pearson Correlation coefficient (R) on common proteins (N) inscribed in scatter plots.

3) Description to supplementary data

Supplementary Data 1:

Supplementary Data 1-Overall description of the dataset	
**sheet name**	**Description**
sheet 01_LFQ-L	LFQ-Light (LFQ-L) lg2 Intensity of individual samples with group summary statistics (outcome, time and combined)
sheet 02_SILAC	Proteins with SILAC ratios (normalized ratios L/H and lg2 transformed)
sheet 03_LFQ-H (SILAC) variance	Robustness of SILAC proteome standard: LFQ-H of 153 proteins with higher than 5% coefficient of variation (CV) across all samples
sheet 04_Venn LFQ-L_SILAC	Venn comparing LFQ-L and SILAC identifications
sheet 05_Enrichment	Enrichments (Metascape Top GO and celltype)
sheet 06	GOCC-categorization tables
sheet 07a-j	Summary statistics on GOCC categories
sheet 08	Individual sample and groups-summary statistics on proteins annotated to Vesiclepedia

Supplementary Data 2:

Supplementary Data 2-Qualitative analysis of EV proteomes	
**sheet name**	**Description**
sheet 01a_Venn_outcome	Venn diagrams of overlapping and unique proteins in PHLF, noPHLf and ALPPS
	Identifications were filtered for 3 (LFQ-L, with mean +/- sd lg2) or 2 (SILAC, with mean +/- sem silac-ratios) values
	LFQ-L proteins appear in filled color (PHLF-red, noPHLF-green, ALPPS-yellow) and SILAC in font colors
sheet 01b_Enrichments_outcom	Source data for Enrichr-Msig-tables and Metascape PPI-Network module-analysis
sheet 01c_Keywords	Uniprot Keywords categorization "Localization", "function" of PHLF or noPHLF+ALPPS only proteins (merging PRE, POD1+POD5)
sheet 02a_Venn PHLF	Venn diagrams for PHLF after filtering for 2 valid values for each timepoint (PRE-POD1-POD5)
sheet 02b_Venn_noPHLF	Venn diagrams for noPHLF after filtering for 2 valid values for each timepoint (PRE-POD1-POD5)
sheet 02c_Venn_ALPPS	Venn diagrams for ALPPS after filtering for 2 valid values for each timepoint (PRE-POD1-POD5)
sheet 03_TOP5_only	Only proteins with highest abundance (Top5)

Supplementary Data 3:

Supplementary Data 3-Comparative statistics on outcome	
**sheet name**	**Description**
sheet 01_overlap_Sig_Top20 regulated	Overlap of all significantly regulated proteins in sheet 03a, 04a, 05a and separte tables of Top 20 up+downregulated proteins
sheet 02a_HM_GO_Top20	Biological interpretation (Top 20 GO-Terms) of significantly regulated proteins (multiple list Metascape) of PHLF vs noPHLF and ALPPS
sheet 02b_PHLF UP	Biological interpretation (Top 20 GO-Terms) of significantly **UP**-regulated proteins of PHLF vs noPHLF or ALPPS (separate list Metascape)
sheet 02c_PHLF DOWN	Biological interpretation (Top 20 GO-Terms) of significantly **DOWN**-regulated proteins of PHLF vs noPHLF or ALPPS (separate list Metascape)
sheet 03a_PHLF vs noPHLF	Student´s Ttests on median normalized lg2 SILAC L/H ratios
sheet 03b_PHLF-noPHLF_Enrichments	Biological interpretation (Heatmap of Top GO-Terms and PPI Networks) of significantly regulated proteins (Metascape)
sheet 04a_PHLF vs ALPPS	Student´s Ttests on median normalized lg2 SILAC L/H ratios
sheet 04b_PHLF-ALPPS_Enrichments	Biological interpretation (Heatmap of Top GO-Terms and PPI Networks) of significantly regulated proteins (Metascape)
sheet 05a_moPHLF vs ALPPS	Student´s Ttests on median normalized lg2 SILAC L/H ratios
sheet 05b_noPHLF-ALPPS_Enrichmer	Biological interpretation (Heatmap of Top GO-Terms and PPI Networks) of significantly regulated proteins (Metascape)
sheet 06_SILAC_outcome_all data	Merged list of all pairwise comparisons with significant (p<0.05, FC<0 or FC>0) and only proteins annotated (+)

Supplementary Data 4:

Supplementary Data 4- Comparative statistics on PRE	
**sheet name**	**Description**
sheet 01_PRE.PHLF vs noPHLF	Student´s T-tests on median normalized lg2 SILAC L/H ratios
sheet 02_PRE.PHLF vs ALPPS	Student´s T-tests on median normalized lg2 SILAC L/H ratios
sheet 03_PRE.noPHLF vs ALPPS	Student´s T-tests on median normalized lg2 SILAC L/H ratios
sheet 04_GO-Top20	Top 20 enriched pathways of significant up or downregulated proteins, only proteins included (Metascape)
sheet 05_Venn diagram regulated	Intersection of UP and DOWN regulated proteins in comparisons PHLF versus noPHLF or ALPPS
